# State of the art of the literature on definitions of self-criticism: a meta-review

**DOI:** 10.3389/fpsyt.2024.1239696

**Published:** 2024-02-19

**Authors:** Vittoria Zaccari, Francesco Mancini, Guyonne Rogier

**Affiliations:** ^1^ School of Cognitive Psychotherapy, Rome, Italy; ^2^ Department of Human Sciences, Guglielmo Marconi University, Rome, Italy; ^3^ Department of Educational Sciences, University of Genoa, Genoa, Italy

**Keywords:** self-criticism, transdiagnostic factor, theoretical models, theoretical perspectives, meta-review

## Abstract

**Background:**

Several authors have developed important theoretical models on an important transdiagnostic factor in psychopathology: self-criticism (SC). Currently, there are substantial variations in the theoretical definition of SC. The lack of awareness of similarities and differences between models may in turn impact the comparison between empirical results, limiting their clinical implications.

**Purpose:**

The purpose of this study was to identify current trends in the field of SC and to explore whether these were approached and shaped by different conceptualizations of SC.

**Methods:**

Core components of the most influential models of SC were identified. A meta-review was conducted searching for systematic reviews and/or meta-analyses in the following databases: PsycINFO, PsycARTICLES, MEDLINE, Scopus, Web of Science, and PubMed (all years up to 28 April 2023).

**Results:**

Contributions were heterogeneous with respect to the definition of SC and the theoretical framework. Almost all systematic reviews poorly addressed the multidimensionality of SC. In addition, discrepancies between the definitions of SC provided and their operationalizations emerged.

**Conclusions:**

The lack of dialogue between the different theoretical perspectives emerged from key contributions in the field of SC. Potential research questions to answer to stimulate this dialogue are proposed.

## 1 Introduction

### 1.1 Self-criticism as a transdiagnostic feature

Self-criticism (SC) is one of the transdiagnostic factors that has received increasing attention in recent years, with several authors developing theoretical models and studying its implications in psychopathology and psychotherapy. Indeed, over the last 20 years, there has been a growing interest in the so-called transdiagnostic dimensions in clinical research and an explosion of empirical contributions on the topic thanks to both the development of measurement tools and the dimensional approach to psychopathology (1, 2) that emphasized the role of autonomous dimensions that can be present in different nosological entities and considered along a continuum of severity or intensity (3). In the clinical field, with transdiagnostic phenomenon, we mean a mechanism that is present transversely in different disorders and that represents a risk or a maintenance factor for the disorder. Briefly, this logic is based on the idea that many disorders share common etiological and maintenance processes, and cognitive, affective, interpersonal, and behavioral characteristics (1, 4–6).

For instance, the theoretical trend that pays a greater attention to transdiagnostic factors in psychopathology (7, 8) is evident in the Diagnostic and Statistical Manual of Mental Disorders, Fifth Edition (DSM-5; 9), which places much emphasis and gives centrality to the transdiagnostic dimensions (e.g., rumination, perfectionism, SC, interpersonal dependency, and guilt proneness). In addition, research in psychotherapy is more interested in identifying the trans-therapeutic factors predicting treatment outcomes of many psychopathological conditions (10; 11–13).

Relative to SC, several studies suggest its association with an array of mental health problems (e.g., 14–18). Indeed, some scientific literature has documented that SC in psychopathology is a transdiagnostic factor and central phenomenon in several psychopathological disorders, accounting for their development and maintenance (19, 20). Furthermore, SC is considered a negative outcome factor in psychotherapy (21, 22). In fact, it is documented that individuals with high levels of SC often obtain little benefit from psychotherapy and are more resistant to treatment (23, 24).

A core topic related to SC is depression, with influential contributions arguing that self-depreciation and criticism are closely related to this disorder (25–27). However, clinical research has shown its link with other psychopathological conditions, too (22).

Although to date, SC is generally considered to be transdiagnostic, as it is present in various clinical profiles and considered a maintenance factor of suffering, its status as a cause or as a symptom of specific clinical profiles (28) has not yet been clarified. Several authors agree that certain forms of SC have multiple functions and effects, including acting as vulnerability factors, accentuation factors of symptom presentation, or hinderers and inhibitors of psychological changes (22, 29–31).

Furthermore, the debate regarding the multidimensionality of SC is also open. Different theoretical models have been developed, which conceptualize SC as a personality trait (i.e., a pervasive and stable propensity to criticize the self) (28, 32), a maladaptive personality characteristic (33), a “mode” (34), a “multiple” (35), or a vulnerability factor (28), whereas others describe it as a coping process (36) or a manifestation of perfectionism (37, 38). This diversity of clarity calls into play another important issue concerning the overlapping of the construct with perfectionism (38–40), rumination (30, 41), or lack of self-esteem (28). Several authors argue that self-critical perfectionism equates to SC and that associated measures can be used to detect the same process (28, 38), similarly for self-critical rumination (41). This overlap also often emerges in SC measurements (30). In that regard, the current scientific literature often highlights the importance of a fundamental challenge for the study of SC: the heterogeneous nature of the construct is often observed (22).

Of note, SC has various forms and functions and does not always evolve into psychological suffering (35). Indeed, SC does not always assume a maladaptive value and lives on a continuum from adaptive to maladaptive aspects of experience (19, 21, 42). According to Gilbert, SC is not a single process but has different forms, functions, and underpinning emotions (35, 43). SC is potentially helpful and a process of fundamental importance for the individual, as it is a self-correcting and a self-monitoring mechanism. In fact, SC is a conscious evaluation of self, providing adaptive feedback to the individual (e.g., promotes reflexivity, or the monitoring of thoughts, emotions, behaviors, or desirable goals). Gilbert clarified that SC has a self-monitoring function that helps people become aware of the need to modify their behavior and facilitate successful goal pursuit (35, 43). Gilbert (43) argued that self-monitoring and self-correcting can be very helpful until they take on a hostile emotional connotation. The difference between adaptive and maladaptive forms of SC would thus be related to the motivation and emotional tone of the critique (35, 43).

### 1.2 Definition of self-criticism

In the last 70 years, the literature on SC has received increasing attention in psychopathology and psychotherapy research (22), even though the phenomenon of SC has already been treated and discussed by Greek philosophers (44). SC began to get clinical attention with the advent of psychoanalysis. Since then, many theorists have emphasized that SC-related feelings and cognitions are important components of psychopathology.

Freud (45), other psychoanalysts (e.g., 46), and cognitive–behavioral (25) and humanistic–existential theorists (47, 48) presented different conceptualizations of SC (44). All the authors, albeit from different theoretical positions, considered the processes of SC in terms of self-judgment and negative cognitions about the self as important factors in psychological suffering.

SC generally refers to the tendency to negatively judge one’s actions, thoughts, and one’s person, typically involving feelings of worthlessness, inability, and inadequacy (27, 28, 35, 49). SC, through a process of self-scrutiny, negative self-evaluation, self-judgement, and self-talk (28, 35, 49, 50), which involves negative emotional reactions such as shame, anger, guilt, and self-loathing (51) and beliefs that one’s/other’ expectations and personal standards have not been met, elicit experiences of disapproval and criticism (21, 28, 33).

Although, generally, the authors agree on the fact that SC typically implies a negative internal dialogue and a negative and/or harsh judgment on the self (28), to date, a plurality of definitions of the construct exists (30). It is interesting to note that the scientific literature offers different descriptions and definitions of the SC constructs underlying its multidimensional nature. The plurality of SC definitions may be connected to the different theoretical models developed, and this plurality of definitions also reflects the overlap of SC with other constructs.

### 1.3 Why we need an umbrella review of self-criticism

Considering the different conceptualizations of SC and its multidimensional nature, it is important to seek an articulated vision of its different forms and functions that may be linked to different psychopathological manifestations. This is likely to stimulate future advances in scientific research. It is important for clinicians and researchers to have an articulated vision of the different forms and functions of SC—that is of its multidimensional nature—that may be linked to different psychopathologies. They would benefit from reaching a clear view of the theoretical background shaping the current trends of research. For instance, this would help in the interpretation of empirical results, facilitating their contextualization and their comparison according to the methodology used. Similarly, awareness regarding the plurality of definitions should reduce misunderstanding in dialogues between clinicians referring to different models.

Considering these premises, the primary goal of the present work was to explore which conceptualizations and facets of the SC construct have been shaping the research field trends and which remain at the sidelines. To reach this aim, we needed to grasp the complexity and the multifaceted nature of SC, carrying out a brief examination of the main theoretical models of SC. Of note, the aim of this preliminary work was not to exhaustively review theoretical contributions on SC, but rather identify the core components of the most influential models that have been used in the recent decades of empirical research. Afterwards, we conducted an umbrella review, collecting highly influential field publications (i.e., systematic reviews) and examined which theoretical components these included in both their rationale and methods.

The term umbrella review (or meta-review) is used to describe a review of systematic reviews or meta-analyses. This methodology allows for the summarization of information and findings from multiple systematic reviews of the literature, facilitating the review and comparison of available results that may have an important impact on future research. Umbrella reviews have been increasingly used in recent years, with some authors stating that these may be considered among the highest levels of evidence available in scientific research (52). This type of contribution may also address broad research questions, such as discussing or comparing different research findings or defining variables of specific interest (53–55).

### 1.4 Conceptual models of self-criticism

According to the Freudian model, SC consists in abnormal levels of morally based self-disregard. This would be due to the predominance of a narcissistic object choice that would lead to a regression causing an identification of the ego with the lost object (“The shadow of the object fell upon the ego,” 45, p. 249). Consequently, the conflictual relationship between the ego and the object would be transformed into an ambivalent relationship between two distinct parts of the self: the sadistic and highly critical part and the victimized part.

Another theoretical framework that has contributed to the development of the SC construct belongs to the tradition of cognitive psychotherapy (25). Beck’s theoretical conceptualization of depression asserts the role of a “negative triad” characterized by automatic negative thoughts about the world, the future, and the self. Beck et al. viewed negative thoughts about the self as self-critical cognitions and beliefs and thus as SC. These negative thoughts about self-represent self-critical cognitions and beliefs. Beck et al. considered SC a pattern that links cognitive, affective, motivational, behavioral, and physiological processes. According to this conceptualization, negative thoughts about oneself are the result of an underlying core schema triggered by specific stimuli or situations (28, 34). In the cognitive model, SC has been considered a stable personality variable (25) and a transitory cognitive phenomenon (56–58).

#### 1.4.1 Blatt’s model

In contrast with the Freudian and post-Freudian conceptualizations of SC that posit that the origin of SC will be found in an existing conflictual relationship, the contribution of the ego-analytic tradition points out the developmental vicissitudes related to the need to meet parental standards. An influential model is the two-polarity model of personality development and psychopathology (49, 50).

Blatt and colleagues developed a theoretical proposal framing two forms of depression into a comprehensive theory of development, personality, and psychopathology (27, 49, 50, 59). The core idea is that self-definition (i.e., one’s sense of self) and relatedness (i.e., one’s sense of relationships with close others) are fundamental dimensions of personality development and core psychological factors accounting for both healthy functioning and the development of psychopathology. These dimensions would develop across the lifespan in a dialectical interaction with each other and significant life experiences.

According to Blatt (59), personality organization structures differ according to interindividual differences between these two polarities. A predominance of the self-definition dimension at the expense of the relatedness one would lead to an introjective organization of the personality, whereas the inverse pattern would be associated with an anaclitic organization of the personality. In particular, the introjective organization is characterized by an internal orientation toward oneself, a high focus on internalized personal values, and a related fear of not meeting one’s high standards. The experience of individuals with introjective organization is dominated by a sense of guilt, inadequacy, self-criticism, perfectionism, and self-punitive attitudes. In contrast, anaclitic organization of the personality is characterized by an external orientation, a tendency to seek security and satisfaction in external relationships, dependency on others for emotional support, and fear of rejection or abandonment. The experience is dominated by feelings of unworthiness, fear of being rejected, incompleteness, shame, feelings of emptiness, and inadequacy (49, 50). According to the type of personality organization, individuals would be diversely vulnerable to specific psychopathological symptoms. In this framework, SC is considered a process underlying self-definition. A delay or deficit in the development of self-definitions can lead to elevated levels of SC, organizing into a personality style.

According to Blatt (59), this is likely to occur after cases of early experiences that undermined the development of autonomy because of excessive physical control, performance expectations, and parental criticism. The child would both experience approval and acceptance as being contingent on meeting strict standards and shape their behavior according to the fear of loss of approval and acceptance. These experiences can lead to a deficit in self-definition and a highly self-critical personality style in which there is an excessive need to establish, confirm, and maintain personal status and positive perception of the self from significant others (60). These developmental deviations would lead to increased vulnerability for mental disorders characterized by feelings of failure, worthlessness, criticism, and guilt (27). This contribution therefore conceptualizes SC as a clinical indicator of an introjective type personality organization characterized by a disposition to have a punitive attitude toward oneself when the standards are not perceived as met; the attitude’s etiological roots can be traced back to relational experiences between parents and children characterized by criticism and punishment (28).

In addition, Blatt (32) distinguished two types of SC, with comparative SC being a proneness to compare own characteristics with those of others (i.e., perceptions of hostility and criticism from others) and internalized SC resulting from the comparison with one’s own ideal (i.e., feeling below one’s ideals). Blatt conceptualized SC as an excessive consideration of the self-definition and defined it as a personality weakness centered on a concern for success and self-esteem causing in subjects with high self-criticism feelings of failure, guilt, inferiority, and shame (49, 51).

Finally, this theoretical approach conceptualizes SC as a trait state, considering the self-critical representation as stable, although it is possible that it manifests as a state in relation to the present mood or external factors (61). Regarding this point, an interesting day diary study using state and trait measures of SC with a sample of community participants supported the idea that SC should be conceptualized as a variable both permanently available and transitorily accessible (62).

#### 1.4.2 Shahar’s theoretical model

Another model that originated from psychoanalytic and psychodynamic theories and formulated by Shahar and Henrich, (63); Shahar (28) is called the axis of criticism model (ACRIM). Its theoretical conceptualization starts from tension between authenticity (A; our inherited potential, or “true self”) and self-knowledge (SK; what we think or know about ourselves). According to this model, the origins of SC lay in parental criticism through their critical expressed emotions and in the failed attempt of the child (and later of the adolescent and then adult) to develop their true self through A and SK. This contribution therefore stresses the role played by childhood adverse experiences in the development of SC (28, 64–67). According to this perspective, greater SC, which makes the individual vulnerable to maladjustment and the onset of psychopathological disorders, results from a lack of A and SK and from experiences of criticism expressed by significant others (15, 68, 69).

Additionally, the ACRIM emphasizes the role of the social ecology within which individual SC is structured. Shahar (28) conceptualizes SC as a result of a network of the individual’s social relationships that follow one another over time, reinforcing SC and increasing vulnerability. Specifically, he argues that SC is structured over the evolutionary span as a result of interpersonal exchanges—first with parents, then with other family members, with peers, and last with teachers—all characterized by criticism, comparison, and rejection. Shahar (28) claims that SC constitutes “a distorted form of self-knowledge that ultimately drives a wedge between an individual’s evolving authenticity and accurate self-knowledge” (p. 92). In particular, the model argues that SC manifests through a dialogue between two parts of the self, in which one part conveys messages of malice, deficiency, and inadequacy that affect the other part. According to this model, SC is characterized by rigid requests for perfection in performance and reactions of hostility and derogation when perfection inevitably fails to materialize. Messages would be characterized by judgmental, derogatory, and harsh tones and would lead to the development of generalized negative beliefs about one’s own nature (e.g., talents, interests, inclinations). These definitive judgments about oneself would prevent one from realizing one’s authentic self. This self-critical dialogue automatically generates emotions (e.g., shame, sadness, and anger) while precluding the activation of positive emotions (e.g., pride or curiosity) that could lead to a process of positive self-examination. According to Shahar (28), cognitive (beliefs about oneself) and emotional (experienced negative emotions) attitudes would consolidate distorted self-knowledge and reduce confusion about one’s identity: “When I am self-critical, at least I know who I am. I am not confused about myself anymore. I have an identity. I am a (bad, deficient) person among people. To relinquish this certainty is to fall back into unbearable confusion” (p. 78).

Importantly, Shahar (28) theorized that a core aspect of SC is its active nature. Indeed, SC has a negative impact in social functioning contexts, generating negative life events and a lack of social support. This can fuel emotional distress, in turn culminating in greater SC. This process would be configured as a vicious circle called a “self-critical cascade,” with SC emerging from negative environmental conditions and leading to emotional distress, which culminated in heightened SC.

#### 1.4.3 Gilbert’s theoretical cognitive-evolutionary model

More recently, Gilbert (70) conceptualized SC through a cognitive-evolutionary lens, arguing for its connection with the motivational system for competition and social rank (31) at the expense of the activation of the cooperation motivational system, accounting for sharing, care-seeking, and caregiving. According to this perspective, two forms and functions of SC can be differentiated according to the presence/absence of the hostile component. In cases of failure, felt frustration may elicit non-hostile SC regarding one’s own performance, leading to feelings of inadequacy that in turn can be adaptive in pushing individuals to self-improve to reach their goals. In contrast, hostile SC targets the self rather than the performance and does not offer perspectives for repairing failures. In this case, the inner dialogue includes a part of the self that feels hatred, disgust, and contempt toward another part of the self that reacts with intense feelings of shame.

The feeling of shame is a key component of the victimized self in the theory of Gilbert (70). According to the author, hostile SC has an evolutionary function that is to protect the individual from external threat. The threat protection system historically consists of two main behavioral correlates that are fight and flight. With the social changes in our societies, placing emphasis on the adaptive value of social connection rather than dominance, reaching a dominant status through physical competition for natural resources has been replaced by the competition for social prestige. Therefore, individuals would no longer physically fight to suppress rivals but instead express disgust and contempt to marginalize others. Complementarily, individuals in a subordinate position would no longer feel fear facing threatening situations; they would feel shame. These new social roles and competencies would in turn have shaped internalized interpersonal schemas that would be used in self-evaluations. As a result, “a dominant–subordinate self-to-self relationship can indeed be acted out internally” (35, p. 33). Of note, these theoretical assertions were empirically supported by recent neurobiological findings evidencing that SC is associated with the activation of the neural substrate of the threat motivational system (71).

Regarding the inter-individual differences in SC, the discussed framework suggests that a key explanation may be found in the early relationships with caregivers. Gilbert et al. (35) suggest that childhood experiences of dysfunctional care may lead to an overdevelopment of the threat protection system, associated with social ranking, anger, anxiety, and disgust, and an under-development of the soothing system that accounts for affect regulation and is fostered by positive emotions experienced during positive and secure affiliative interactions with significant others (19, 72). Specifically, a developmental environment perceived as unpredictable and threatening, or where the feeling of lovability is rarely experienced, would lead the child to develop a proneness to harshly monitor and punish their own errors to maintain high levels of internal locus of control.

Of note, these assertions mostly converge with the understanding of SC offered by attachment theory (73). The concept of “defensive exclusion” seems especially useful in understanding how and why children develop a proneness to SC. The framework asserts that children struggling with caregivers prone to negatively react to attachment signals (e.g., rejection, criticism) would learn that showing physical and emotional distress to the attachment figure(s) would result in increased emotional arousal. Internal and external triggers activating the attachment system would be excluded defensively out of consciousness to avoid an escalation of distress. This process can shape the development of personality, with the individual developing self-reliance beliefs, high expectations for the self, and poor tolerance of one’s own failures (74). In line with these theoretical expectations, most studies have shown that the avoidantly attached are typically highly self-critical (75).

Another explanation emerging from attachment theory (73) is related to the proneness of abused children to self-criticize in reaction to blameful caregivers’ behaviors. This would be explained by the primary goal of the child, which is to preserve their perception of a safe environment. In other words, this child assumes the responsibility for the harmful behavior of the caregiver, despite being costly in terms of self-worth, which guarantees the perception of a benevolent and predictable world. Again, the relatively large amount of literature documenting the association between early childhood trauma, anxious attachment, and proneness to SC appears to support the contribution of attachment theory to the field (75). Interestingly, the approach of Gilbert adopts several of the attachment theory concepts, as the role of the attribution of the fault is considered crucial in the development of SC. Specifically, the author argues that the shameful reaction of the self—from which originates SC—is characterized by the attribution of the fault to the self. In contrast, in cases where the critics are perceived as unjust, the individuals would feel humiliation and anger and would develop a desire for revenge (76).

### 1.5 Rationale and research question

The literature overview provided in *Introduction* highlights some variations in the theoretical definition of SC. Another observation consists of the lack of dialogue between the different theoretical perspectives, although to date, it is considered an important transdiagnostic factor (central to several conditions of psychological suffering) and a trans-psychotherapeutic negative outcome factor. Briefly, it appears that despite several areas of convergence between approaches, each model stresses different components. To explore the way the plurality of approaches to SC shapes current trends of research in the field, we conducted an umbrella review of contributions focused on the topic of SC. Indeed, an optimal approach to grasp the current trends in hot-topic research consists of analyzing systematic reviews performed on the topic. These are key contributions because they offer a collection and a synthesis of all the studies carried out on a specific topic according to a rigorous methodology. These studies benefit from a research methodology that has been widely used in the last decade; they offer the advantage of impartially considering the conflicting data available in the literature. These contributions are especially influential in that they provide indications that both shape the direction of future research trends and influence clinical practice. Therefore, analyzing and summarizing the systematic and meta-analytical research carried out was thought to be a good starting point for encouraging epistemological reflection. In this regard, we aimed to carry out a meta-review of the theoretical and empirical literature published on SC to examine the ways these contributions addressed the definition of SC and the theoretical framework used, the multidimensionality of SC, the methodological features, and the consistency between the various SC definitions and their operationalizations.

This review was thought to be relevant for clinicians and the scientific community that sought light on how knowledge in the field has advanced and in which directions the research is moving. The primary aim of this meta-review was to both identify current trends in the field of research of SC and explore how these approached the plurality of theoretical models.

### 1.6 Comparison of theoretical models

Examining the degree by which key contributions (i.e., systematic reviews) on the topic addressed these theoretical issues needed a preliminary identification of core features of the main theoretical models of SC. Therefore, a critical summary was conducted to compare the way the components of SC have been theorized across the most influential models. This analysis was pursued with the aim of analyzing the components of SC from the various theoretical models available and observing how they are addressed in the systematic reviews. Our purpose did not require finding a single model of SC, rather it was thought useful for highlighting areas of convergence and divergence between the available theoretical models.

Over the years, all theoretical frameworks reviewed here have been used in many scientific studies to test whether SC is associated with psychopathology and/or poor therapeutic results. On the one hand, the burgeoning of these theoretical proposals boosted empirical investigation of the topic, leading to relevant knowledge in the field of clinical psychology. On the other hand, a closer look at the state of the art in the field of SC quickly evidenced that these frameworks often developed independently without specifying their differences with the other approaches. Notably, results from different theoretical contexts can be difficult to compare, limiting the generalizability of the observations made by single investigations.

Currently, valuable theoretical models of SC have been developed in the literature, as illustrated above. However, no current review had attempted to systematize SC key features. To critically compare theoretical proposals is, however, crucial for evidencing both areas of convergence and divergence and aspects that are not well clarified and deserve further attention. In the field of SC, a valuable effort was made by Shahar (28), who summarized models of SC according to several aspects (i.e., definitions of SC, strengths, and limitations) while highlighting what kind of definition was provided by individual authors and the respective, theorized etiological roots of their version of SC. However, Shahar’s summary missed some important issues, such as the “target” of SC, the emotional valence or tone of the criticism, and the emotional reactions of the self-criticized individual. For instance, some authors place the accent on how SC is linked differently to guilt and shame depending on the object of the criticism (e.g., one’s behavior, one’s appearance, sense of self, or one’s motivations or emotions), as self-criticism is not always polarized on the global self (77). The lack of clarity regarding these aspects might impact the conclusions of future empirical studies that could struggle to contextualize their results in wide scientific, panoramic terms.

Specifically, in this work, the following components were selected: the a) etiological factors (i.e., roots and vulnerability); b) target of SC (i.e., the content of the evaluation); c) emotional valence of critics (i.e., the emotional tone of the evaluation); d) emotional reactions of the self-criticized individual; e) psychopathological model (i.e., the psychopathological implications of SC, or SC as a factor of vulnerability to psychopathology or factor in specific clinical profiles); and f) nature of the variable (i.e., the negative judgement, personality trait, or self-representation). Next, for each of these components, we provide a brief illustration of the convergence and divergence areas between theoretical models and gray areas that were insufficiently addressed by current models.

The components that we coded were developed through a top–down and bottom–up approach. Two authors [blinded for review] independently listed core theoretical components that they expected to find in theoretical models of SC. Afterwards, a summary for each of the main identified models (also consulting 28) was created in collaboration. After the extraction of information and the coding process, discussions between authors led to the proposal of adding components (i.e., when relevant aspects of theories were not sufficiently documented by the previous categories), removing components, and/or modifying labels of categories. Results of this process are summed up in **Table 1**.

**Table 1:** Main theoretical components of the current available framework on self-criticism.

Etiological factors	Evaluative features	Emotional valence of critics	Target of self-criticism	Emotional reactions of the self-criticized individual	Nature of the variable	Psychopathological model
Blatt and Luyten et al. (49) Blatt et al. (50)						
- Excessive parental physical control - Excessive parental performance expectations - Excessive parental criticism - Delays or deficits in the development of self-definition	- Self-scrutiny - Self-stance - Comparative with others - Comparative with one’s own ideal	- Punitive - Harsh - Hostile - Deprecative	- Personal characteristics - Behavior	1. Fear - of failures - of being criticized 2. Feelings of unworthiness - inferior to one’s own ideals - inferior to others - failure 3. Guilt 4. Shame	- Personality trait - State	- Introjective personality (depression) - Vulnerability to psychopathology
Shahar (28)						
- Excessive parental criticism - Excessive parental expressed emotions - Social ecology - Delays or deficits in the development of the true self through authenticity and self-knowledge	- Self-scrutiny - Self-evaluation - Demanding	- Punitive - Harsh - Hostile - Deprecative - Uncompromising - Derogatory - Cruel	- Behavior - Whole self	1. Feelings of unworthiness - feeling deficient - inadequacy 2. Feeling bad - shame - sadness - guilt - anger - wickedness	- Personality trait - Relationship between two parts of the self - Active nature	- Vulnerability to psychopathology
Gilbert et al. (35)						
- Dysfunctional care in childhood - Overdevelopment of the threat protection system - Under-development of the soothing system - Social mentality	- Self-judgement - Attacking self	- Hostile - Coercive - Hateful - Disgusted	- Behavior - Whole self	1. Feelings of inadequacy 2. Shame	- Schema - Inner dialogue between two parts of the self	-Vulnerability to psychopathology

#### 1.6.1 Etiological factors

Of note, all models appeared to agree in considering the crucial role of early childhood experiences in shaping the propensity to SC (27, 28, 35). Specifically, Blatt stressed the components of physical control and high parental expectations, Shahar pointed out the role of expressed emotions and parental criticism, whereas Gilbert depicted a caregiver with low empathy being either disengaged, controlling, preoccupied or cold, anxious, or critical. Furthermore, only Shahar (28) model, in relation to vulnerability, emphasizes the concept of social ecology within which individual SC is structured and kept active.

Noteworthy, models appeared to focus on different dimensions or motivational systems involved in the development of SC. Whereas Blatt and Shahar mainly refer to delays or deficits in the development of self-definition, Gilbert postulates an excessive overdevelopment of the threat protection system and underdevelopment of the soothing system. In addition, as detailed above, Gilbert explicitly refers to the role of the attachment system and its vicissitudes experienced during childhood. In addition, according to each author, the nature of childhood trauma shapes the “theme” of SC that in turn would be associated with a distinct type of shame (76, 78, 79).

#### 1.6.2 Target of self-criticism

Importantly, most models appeared to agree that the “victim” of the criticism is the behavior. Particularly, the Blatt and Shahar models focus on performance, in which there is a comparison with one’s ideal and/or with the others’ characteristics. These authors emphasize the importance of comparison with others in SC. Going further, Blatt specifies that personal characteristics, too, can be the target of the SC process, and Shahar also refers to the “whole self.” Similarly, Gilbert argues that the object of the self-hatred form of SC is the whole self rather than a single behavior.

#### 1.6.3 Emotional valence of critics

Regarding the description of the evaluation process, the models of Blatt and Shahar place emphasis on the nature of SC as one of negative self-evaluation, a self-stance, and/or self-scrutiny, whereas Gilbert provides a cognitive description of the process (“self-judgment”). In addition, Blatt introduces the notion of a comparative process (both toward personal standard and others) that overlaps only partially with the demanding nature of the self-requests described by Shahar.

Importantly, these aspects are not cited in the conceptualization of Gilbert, who instead stresses the aggressive nature of the process, described as an “attack.” Furthermore, Shahar (28) provides further specification of the description in which SC is seen as a “forceful prosecutor in a trial, against whom the best we can do is try and marshal some strength to defend ourselves” (p. 79).

Noteworthy, the only emotional valence on which there appeared a consensus consists of the hostile component. This seemed relevant, as Gilbert stresses the idea that the hostile and harsh emotional valence of critics is the signal that (pathological) SC is rooted in the motivational threat system and is not aimed at self-correction like negative self-evaluations that do not have a harsh emotional tone (43). Empirically, Whelton and Greenberg (51) observed that the quality of the “emotional texture” of SC differentiates individuals with high versus low levels of proneness to SC, and reactions to SC. Specifically, higher levels of sadness, shame, and unassertiveness were observed among individuals with SC characterized by intense contempt and disgust. Similarly, for Firestone (78), the pervasiveness and the intensity of the emotional valence of the critics is a signal of the self-destructive function of SC. In addition, this author observed that the emotional texture of the critics is highly traceable to specific caregivers’ figures, illuminating the etiological factor of the “inner voice” pushing individuals to suicide.

Regarding the specific emotional texture of critics, whereas the descriptions of Blatt and Shahar mostly overlap (i.e., punitive, harsh, deprecative), Gilbert better specified a description evidencing the hateful, disgusted, and coercive nature of critics. Finally, further specificity was offered by Shahar, who stresses the uncompromising nature of SC and its judgmental, derogatory, harsh, and even cruel tone.

#### 1.6.4 Emotional reactions of the self-criticized individual

Conceptualizations appeared to vary in the way they describe the feelings of the individual who is a victim of SC. For Gilbert, the painful feeling of shame is primary. The model of Shahar added other descriptions like feeling deficient, inadequate, or feeling bad, and experiencing emotions like shame, sadness, and guilt. In particular, only Shahar also identifies the feeling of “wickedness.”

Finally, Blatt describes several types of emotional reactions of the individual, including, in some cases, sub-specifications. Indeed, the individual may experience fear (of failure or of being criticized), feeling unworthy (i.e., feeling a failure or inadequate with respect to others or to one’s own standards), and guilt and shame.

#### 1.6.5 Psychopathological model

Of note, regarding this point, only the model of Blatt was developed according to a model of a specific mental disorder (in this case, depression). The models of Shahar and Gilbert define SC as a vulnerability factor for psychopathology but do not refer to a specific class of disorders.

#### 1.6.6 Nature of the variable

Finally, the models did not always appear clear regarding the nature of SC as a psychological variable. For instance, the three perspectives seemed to conceptualize SC as an enduring psychological characteristic (i.e., a “personality trait” for Blatt and Shahar, a “schema” for Gilbert), but only Blatt also explicitly considers SC a potential transitory mental state. Lastly, both Shahar and Gilbert conceptualize the process as the interaction between two parts of the Self, expressed through inner dialogues. In addition, it is only in Shahar’s model that the active and interpersonal nature of SC was conceptualized.

## 2 Method

**Figure 1:**
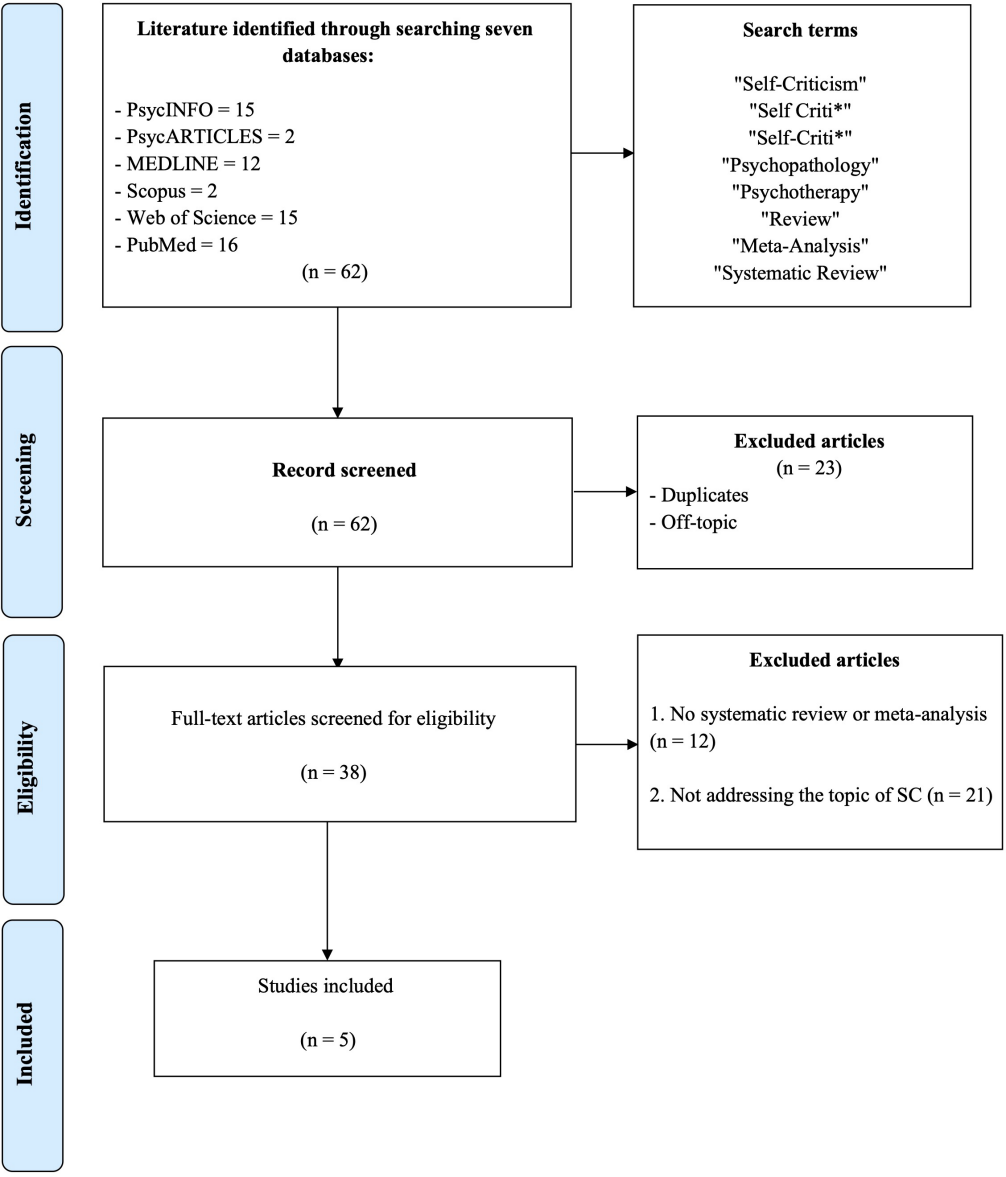
Flow diagram of the search and screening process of contributions included in the meta-review on self-criticism.

A meta-review was conducted according to the PRISMA guidelines for systematic review (80). The process of identification and selection of studies was carried out based on inclusion criteria illustrated in a flow diagram in **Figure 1**.

### 2.1 Search strategy

A search string was initially developed that operationalized our main search questions, which contained search terms divided into three concepts: “self-criticism,” “psychopathology,” and “psychotherapy” (see **Appendix A** ). Six scientific databases were searched, including PsycINFO, PsycARTICLES, MEDLINE, Scopus, Web of Science, and PubMed; we set no restrictions on the year of publication; all dates up to and including 28 April 2023 were fair game.

### 2.2 Selection criteria

The following criteria were selected for inclusion of studies in the meta-review: (1) the study was a systematic review or meta-analysis with a search strategy and inclusion/exclusion criteria clearly described, and (2) the study addressed the role of SC. Exclusion criteria were (1) not being published in an international peer-review journal (e.g., gray literature) and 2) not being a systematic review or meta-analysis (e.g., narrative review, book review, editorial, or comment).

### 2.3 Selection of studies

Two blinded reviewers [blinded for review] independently searched the selected scientific databases. All titles and abstracts were analyzed and screened according to our selection criteria. The search identified a total of 62 records. All studies were evaluated by each reviewer for suitability. Following the removal of duplicates (performed with Zotero software), a total of 38 articles were screened, of which five were included in the meta-review. Any critical issues noted (such as ambiguity in the title and abstract) were addressed by reading the full text of the review article by both reviewers to determine suitability. Both the performed search and extracted results were checked by both reviewers. Any disagreement about the information revealed was resolved through discussions between all authors. After being carefully evaluated and discussed, the articles that met the eligibility criteria were selected for this meta-review. The identification and screening procedure is summed up in the flow diagram in **Figure 1**.

### 2.4 Data extraction and synthesis

A protocol listing the information for extraction from the contributions was developed specifically for the purpose of this study (see **Appendix B**). The extracted data were collected by the first author and rechecked by the third author. A form was designed to extract the following meta-data of the contribution: authors’ names, year of publication, country, affiliation, funding, published status, review method, and information regarding SC and the study methodology (i.e., aims of the study, research questions, definition[s] of SC, theoretical frameworks cited in the Introduction section, selection of theoretical framework, premise regarding the multidimensionality of the construct, theoretical frameworks cited in *Discussion*, conclusions regarding the multidimensionality of the construct, number of scientific databases, names of databases, search strategy for gray literature, restrictions, keywords operationalizing SC, and selection criteria regarding SC). Data and information selection were done by both authors through an analysis and discussion until a consensus was reached.

## 3 Results

### 3.1 Main characteristics of the studies

A total of five studies were included in the final meta-review. The main characteristics of these studies are displayed in **Table 2**. Three studies were meta-analyses and systematic reviews, and the remaining were only systematic reviews. The publication years range from 2018 to 2021. Four studies were conducted in Europe (22, 31, 81, 83), and one (82) was conducted in the United States.

**Table 2:** Main information extracted from the included contributions.

Authors	Method	Aims	Etiology	Evaluative features	Emotional valence	Target	Emotional reaction	Nature
81	Systematic review and meta-analysis	To estimate the overall effect of self-compassion-related interventions for SC and to investigate the moderating effect of methodological characteristics and SC dimensions	Not specified	- Negative self-evaluation - Self-scrutiny	- Anger - Hate - Contempt	- Inadequacies - Things to improve	Not specified	Not specified
22	Systematic review and meta-analysis	To summarize the associations between SC and psychotherapy outcome and their moderators	Early parent-child relations and life span events determining failures in the self-definition process	- Self-scrutiny - Overly critical evaluations - Self-persecution	- Harsh - Self-bashing - Hostility	- Behavior	- Concerns over mistakes - Inability to derive satisfaction from performance - Feelings of failure - Guilt - Feeling of inferiority - Shame	- Personality variable - Automatic thoughts
31	Systematic review	To summarize and integrate empirical findings in SC research and to identify gaps in the research of SC; to review the relationships between SC and psychopathology and clinical interventions targeting SC	- Parental criticism - Critical expressed emotions - Failures in the self-definition process - Childhood maltreatment	- Self-critical voices	- Hate	Not specified	Entrapment	- Schema - Motivational system correlate
82	Systematic review and meta-analysis	To investigate SC as a transdiagnostic correlate of non-suicidal self-injury and disordered eating; to examine the relation of self-criticism to NSSI and DE and its moderators	Not specified	- High aversive cognitions - Self-critical thoughts	Not specified	Not specified	Not specified	Not specified
83	Systematic review	To explore longitudinal associations between SC and psychopathological symptoms among students	Not specified	- Self-judgment - Self-scrutiny	- Harsh - Punitive	-Achievement- related events	- Negative affect	Not specified

None of the included studies were conducted by authors affiliated with scientific societies reporting a specific psychological orientation. In addition, only two studies received funds for publication (22, 82).

All studies clearly stated aims and research questions. The systematic reviews generally placed emphasis on summarizing existing knowledge about the associations between SC, psychopathology, and psychotherapy outcomes and the nature of potential moderating variables of these associations.

In particular, Löw et al. (22) meta-analyzed available data on the association between SC and psychotherapy outcomes and tested the moderating role of several variables. Along the same lines, Wakelin et al. (81) meta-analyzed then–current results to explore if different forms of SC affect the relationship between therapy and SC-related outcomes, and which variables moderate these links. The first published contribution (83) collected evidence from prospective studies to test the hypothesis that SC predicts higher levels of psychological symptoms among students. Extending this work, Werner et al. (31) critically summarized empirical findings from SC research and evaluated whether different forms and functions of SC are related to psychopathological outcomes and the extent to which different psychotherapy approaches reduce dysfunctional forms of SC. Lastly, Zelkowitz and Cole (82) adopted a more focused approach framed by the idea that SC should be considered a transdiagnostic factor accounting for both non-suicidal self-injury and disordered eating. To reach their aim, the authors summarized qualitative studies and meta-analyzed quantitative studies documenting the relationship between SC and both these clinical conditions.

As the main goal of our study was to illuminate how these reviews encountered through a systematic methodology the concept of multidimensionality and the theoretical conceptualization of the construct of SC, we structured our *Results* in four paragraphs, reporting data regarding the a) definition of SC and the theoretical framework used, b) multidimensionality of the SC construct, and c) methodological features and consistency between the SC definition and its operationalization. The decision to describe the studies via these categories was made with the aim of clarifying the theoretical frameworks, the definitions of SC adopted by the scientific community, and the systematic criteria used in order to also analyze how the construct was contextualized.

### 3.2 Definition of self-criticism and theoretical framework used in contributions

Concerning the definitions of the SC construct, only three contributions (22, 81, 83) provide a clear description of SC, whereas the others were almost vague. For instance, in the Introduction of Werner et al. (31), an explicit reference to a definition of SC is not provided, with the authors only describing three theoretical models of SC (28, 70, 84) and placing emphasis on vulnerability.

The most frequently cited definitions were those of Blatt and Shahar, followed by those of Gilbert. Surprisingly, this result does not perfectly mirror the trend regarding the illustration of the theoretical model, with only one study mentioning Shahar’s model in its introduction, and with the Blatt and Gilbert conceptualization being equally popular across contributions. Furthermore, no authors cited Shahar’s model when framing their results, with most citing Blatt’s model and the others referring to Gilbert’s theory. Of note, other frameworks were cited by the systematic reviewers that we did not register as official models of SC. Specifically, some contributions refer to the work of Beck (25, 34) or the framework of perfectionism (37).

An additional general observation was that descriptions were sometimes vague and did not provide specifications on the conceptual bonds delineated above. For instance, two contributions do not provide any clear definition of the construct (31, 82). Then, regarding the remaining contributions, as expected, these were quite heterogeneous regarding the descriptions of SC. Of note, the component reaching near consensus was related to the evaluative nature of the SC process, always described with the term “self-scrutiny,” along with negative self-evaluation/judgment.

Only two contributions (22, 31) mention the etiological factors of the theoretical frameworks selected in the review, although they only partially recall the etiology of the models mentioned and reviewed above. In the overviews, the authors provided of the construct; the emotional valence of the critics was not always clearly depicted. McIntyre et al. (83) and Löw et al. (22) refer to the harsh and punitive characteristics of SC, whereas the hateful and aggressive nature of SC was indirectly suggested in Wakelin et al. (81) and Werner et al. (31).

Of note, other authors stressed the hostility component of SC (22). In addition, the emotional reaction of the criticized are depicted in ways that were not conceptualized in the framework we reviewed. For instance, only two contributions cited the anger or contempt valence of the criticized (81) and feelings of failure, guilt, inferiority, and shame (22).

The “target” of the critic is not made explicit in all contributions. Moreover, the contributions differ in citing the nature of the components that are the target of SC processes, being either the behavior, the reaction, or the failures of the self. Wakelin et al. (81) cite inadequacies, while Löw et al. (22), behavior. Contributions that selected a specific theoretical framework (i.e., Blatt’s, now Gilbert’s, model), such as Löw et al. (22) and Wakelin et al. (81), do not mention the characteristics reviewed above in relation to the Blatt and Gilbert model (i.e., personal characteristics, hurting the self).

Lastly, the contributions are not always clear regarding the nature of SC as a psychological variable. Only Löw et al. (22) conceptualize SC as an enduring personality variable or a transitory cognitive phenomena in line with Blatt (33) conceptualization and with the adopted theoretical framework. However, authors also documented SC as a psychological variable related to perfectionism or a maladaptive and pathological facet. Two contributions (31, 82) describe SC as a vulnerability factor, and in particular, Zelkowitz and Cole (82) support the nature of SC as a psychological variable related to self-harm. No contribution citing Gilbert’s or Shahar’s models conceptualizes the process as the interaction between two parts of the self that manifests itself through the form of inner dialogues.

### 3.3 Multidimensionality of the self-criticism construct

Four systematic reviews acknowledge the reader regarding the multidimensionality of the SC construct (22, 31, 81, 83), highlighting different theoretical conceptualizations and the heterogeneity of the construct reflected in the different measures of SC. Only one study (22) highlights the content overlap between SC and the construct of perfectionism.

The conclusions of all studies appear framed in the light of the multidimensionality of the construct, with contributions stressing, in a vague manner, that the presence of different forms of SC are not discriminated against in different forms in relation to psychopathology. However, no study drew conclusions regarding the multidimensionality of the construct. Specifically, no indications are provided on which dimensions of SC were most noted in the studies examined, which ones are more or less involved in clinical profiles, or which ones deserve further study.

### 3.4 Methodological features and consistency between the self-criticism definition and its operationalization

Each contribution searched at least three scientific databases, but only Löw et al. (22) carried out a check of reference lists of included studies and other reviews. All systematic reviewers used search restrictions regarding language and time period, except for the study by Löw et al. (22), who did not specify a period.

In their search-term lists, studies used different keywords to operationalize the SC construct. The only study (Wakelin et al., 2021) that selected keywords referring to a multidimensional conceptualization of SC refers to Gilbert’s theory. Of note, no study used keywords referring to the conceptualization of SC as a personality trait (e.g., “enduring dysfunctional attitude”), despite that some contributions were explicitly framed within the Blatt theory (22) or used the Blatt’s definition of SC (31, 83). Importantly, two studies (22, 83) selected keywords referring to perfectionism, although only Löw et al. (22) explicitly assert a theoretical overlap between SC and this psychological variable.

All studies included quantitative instrument(s) measuring SC in the selection criteria of studies but differed regarding which were considered valid measures. Three contributions (22, 31, 83), together with the definition of SC provided in the introduction, included studies measuring SC tied to Blatt’s or Gilbert’s theoretical proposals.

In addition, Löw et al. (22), congruently with their definition of SC, included studies measuring perfectionism. The most inconsistent links between the theoretical framework selected and the inclusion of tools as valid measures of SC were observed in Wakelin et al. (81). Indeed, they included studies using the not-judge subscale of the Five Facet Mindfulness Questionnaire (85), albeit a critical attitude toward one’s own emotional state was not explicitly stated to be a central feature of SC. In addition, these authors included studies using the self-critical perfectionism subscale of the Dysfunctional Attitude Scale (86), but they did not discuss perfectionism as a dimension of SC. Two other studies (22, 82) included the self-hate scale from the Structural Assessment of Social Behavior (87), Intrex questionnaire, without cohering with the selected theoretical framework. Lastly, the consistency between the list of measures considered valid and the theoretical framework selected by the systematic reviewers was not evaluable in the contribution of Zelkowitz and Cole (82), as these authors did not explicitly select a theoretical framework.

## 4 Discussion

The main goal of this study was to stimulate the burgeoning field of research on SC by identifying current trends. To reach this purpose, we performed an umbrella review, considering that systematic reviews and meta-analyses are key contributions that grasp hot topics in research and are influential in shaping future research directions. Results were analyzed using a critical perspective, extracting information on the way systematic reviews framed each component of the theoretical models related to SC that we identified in *Introduction*.

Before discussing results regarding each of these components, some observations should be articulated regarding the general characteristics of the studies included. Results highlighted that SC has received increasing attention in recent years, being extensively studied in relation to its implications in psychopathology and treatment outcomes. Moreover, it is noteworthy that the selected studies were primarily conducted in Europe, with only one conducted in America and none from other continents. Therefore, our conclusions may be biased, for instance over-representing European cultural factors and psychological perspectives. This highlights the need of additional non-European studies on the topic.

The contents of research questions in the contributions reviewed indicate that SC has been investigated in relation to different clinical profiles, such as mood disorders (specifically depression, depressive symptoms, and suicidality), eating disorders (22, 31, 81, 83), social anxiety 22, 31), obsessive–compulsive disorder, schizophrenia/psychotic disorder, paranoid delusions (22, 81 31), personality disorders (borderline personality disorder; 22, 81), and behaviors like trichotillomania, chronic fatigue syndrome, and stress (22). Systematic reviewers also paid attention to both the existing knowledge on the associations between SC, psychopathology, and psychotherapy results and on the nature of the potential moderating variables of these associations. Our results underline the relevance of the construct involved in a wide range of mental disorders and predictive of therapeutic outcomes. However, this increased interest in SC contrasts with the lack of attention paid to theoretical aspects, as illustrated next.

### 4.1 Definition of the construct and considerations on multidimensionality

As extensively discussed in *Introduction*, despite theoretical models of SC converging on some features of SC, other aspects related to the construct differed across approaches, resulting in a plurality of definitions (30). Importantly, previous contributions argue that the lack of dialogue between models of SC may be partially due to the multidimensional nature of the construct that has not always been sufficiently considered by theorists (22). To understand how these issues have been addressed by research, information was extracted regarding the nature of the definitions of the construct in each study and the consideration authors paid to its multidimensional nature.

First, it was noted that the most frequent definitions of SC were related to the models of Blatt et al. (26) Blatt and Zuroff, (–) Shahar (28) Blatt and Luyten (49) Blatt et al. (50), and Gilbert et al. (35), with various contributions referring to several theoretical frameworks. However, only a few contributions provided a clear definition in their rationale, with most of them offering only vague descriptions of SC (31, 82). In addition, definitions widely differed across studies, despite some areas of convergence such as the evaluative nature of the SC process. On the one hand, this result may suggest a lack of dialogue around the features of SC. Conversely, this may indicate a tacit agreement toward the definition of the construct. However, these results seem to mirror what emerged from a critical analysis of existing theoretical models of SC, that is, a lack of dialogue and integration between the different perspectives on SC. The multiplicity of SC descriptions observed in the included systematic reviews seems, therefore, to support the idea that this lack of dialogue hampers the possibility of clearly delineating the construct investigated in empirical studies. However, it is important to recognize that the analyzed studies included more than one theoretical framework for SC, as an integration of the different existing theoretical perspectives and a univocal conceptualization of the construct is yet to appear in the scientific literature.

In addition, this concern is further emphasized by the observation that most systematic reviews showed a limited congruency between the definition of SC provided in the rationale and the way the construct was operationalized in the choice of search terms and/or selection criteria. Indeed, the theoretical limitations regarding the lack of a clear definition of the construct appear to have impacted the consistency between the rationale and methods used, in turn limiting the usability of results brought by systematic reviews. In other words, the lack of clarity regarding the nature of the construct investigated limits both the soundness of the conclusions drawn by authors and the ability to compare results obtained by different systematic reviews.

Finally, we observed that the issue of multidimensionality of the SC construct has been poorly addressed by systematic reviews. Examining SC as a multidimensional construct may be a useful approach to better define—and consequently operationalize—specific features of the construct, potentially justifying the reference to different and non-integrated theoretical models. However, this opportunity was rarely grasped by researchers, except for Löw et al. (22), who discuss the overlap between SC and perfectionism. Indeed, despite most authors briefly alluding to the heterogeneity of the construct when discussing these results, this was often limited to the observation of the multiplicity of measurement tools. These considerations may have clinical implications related to the heterogeneity in the measurement tools used to identify SC. The tools have been developed according to different theoretical models. For instance, the contents of the items and the type of factors in the Forms of Self-Criticizing/Attacking Reassuring scale (35) and in the Levels of Self-Criticism Scale, comparative SC and internalized SC (88), vary according to their theoretical background. Consequently, specific assessment tools may better assess the quality of SC in specific psychopathological conditions but not in others. Furthermore, clinicians should select the type of measurement tools according to their degree of interest in evaluating etiological factors or the state/trait SC process. In other words, clarifying the specificities of the theoretical background underlying particular assessment tools is likely to help the clinician select the one that is more tailored to their patient’s specific needs. For instance, results were not framed considering this multidimensionality and were not used to better specify which components of SC were more involved in psychopathological or clinical outcomes. In addition, no considerations were provided regarding the dimensions of SC that remained on the sidelines of empirical studies, and directions for future research were not tailored to specific dimensions of the construct.

These considerations highlight the need to answer several still-open questions. Might we create or find a comprehensive approach to SC that keeps multiple definitions together? Might a multidimensional perspective leverage this process? We will argue here that a dialogue between models of SC would help advance the field. Specifically, we propose that the similarities and differences across models may be understood in light of the multidimensional nature of the construct. The perspective illustrated in the next section hopefully helps researchers formulate new working hypotheses regarding why SC can be characterized by different etiological factors, emotional valences of critics, targets of critics, emotional reactions of individuals, and psychopathological implications.

### 4.2 Etiological factors

In the first section of this manuscript, we noted that despite all theoretical frameworks considering crucial the role of early negative childhood interactions with caregivers in determining vulnerability to SC, each of them stressed the role of additional specific types of experiences (27, 28, 35). Of note, we observed that the contributions we retrieved paid little attention to this component, with only two contributions briefly mentioning it in their rationale (22, 31). This might be partially explained in considering the aims of the studies included that mostly focused on the correlates of SC rather than the mechanisms leading to its onset. However, this also highlighted that this area of research is overlooked in the current trends of research. This might be problematic for advancing our knowledge of SC. Indeed, the etiological hypotheses regarding factors accounting for SC development deserve empirical testing to reach a more comprehensive understanding of the construct and to better specify the rationale underlying interventions targeting SC.

Importantly, these considerations question the fact that the etiopathological factors documented now are sufficient to account for the complexity of SC and its manifestations across clinical conditions. We suggest that the different forms of SC may be related to different etiopathogeneses, and the quality of criticism is likely to be different. In other words, specific types of etiological risk factors would illuminate a specific dimension of SC. For instance, the quality of criticism is likely to be different according to the nature of the risk factors, such as traumatic relational experiences, negative parental metacognitions on emotions, extra-familiar adverse experiences, sexual trauma, reactions to dysfunctional resolutions of conflict as phenomenon of the “silent treatment” (89, 90), reject, blaming, hypo- or hyper-responsibility, or the observation of someone else’s guilt or contempt.

The presence of different types or forms of SC might also help explain clinical comorbidities. People self-criticize for different reasons; therefore, the target of the criticism would be different. This is frequently observed in clinical practice. Patients show different qualities or judgment criteria for criticism. We suggest that SC manifests differently in different clinical conditions, which in turn are related to different etiological factors. This also recalls the importance of the contextual specificity in which vulnerability to SC develops.

### 4.3 Target of self-criticism

An aspect that was not clear and divergent across models of SC was related to the target of the SC process. Some authors referred to the “whole self” without further specification; others pointed out an individual’s behaviors or characteristics, whereas others identified an individual’s performances. As expected, the plurality of specifications regarding this component in theoretical models was mirrored in the studies examined. Indeed, this aspect was not specified in the rationale of systematic reviews, and the conclusions drawn did not extend the knowledge regarding this point. For example, moderation analyses have not been performed yet, and this might limit the utility of studies in the field regarding the understanding of the ways SC interplays with other psychopathological mechanisms according to clinical conditions. Indeed, SC may be differently involved in mental disorders according to the type of target involved. Consequently, clinical interventions for SC that ignore accurate considerations regarding which aspect of the self the critics target should not be planned.

It emerged that we need a systematization of the range of components that may be targeted by SC. For instance, the self can be criticized when personal goals are threatened. This difficulty can lead to a criticism aimed at a specific performance (i.e., emotions, conduct, behaviors, thoughts, actions, and omissions) or general personal skills (i.e., global judgment on the whole self). This distinction was previously suggested in the differentiation between behavioral and personological self-blame (91). We suggest that the target of SC should be well differentiated according to several criteria and that the nature of this target is central to identify the different dimensions of SC.

### 4.4 Emotional valence of critics

In our brief overview of the theoretical approaches to SC, we noted that a crucial component of theoretical perspectives was related to the nature of the emotional valence associated with the critical process. Indeed, while the hostile nature of the critics was recognized by all models, these differed in the extent that they stressed specific connotations such as hateful, disgusted, deprecative, or punitive. In the systematic reviews, we found that this aspect was not well specified, with only one study stressing the hostility component (22). This might be a relevant issue because different emotional valences of critics are likely to be associated with different dimensions of SC that in turn may be associated with different psychopathological outcomes and correlated risk factors (31).

Therefore, to date, it is not clear which types of emotional valences characterize various critics, and some connotations are likely to co-occur, while others may differentiate the type of SC dimension. We may, for example, speculate that different emotional valences of criticism, conceptualized as inner critical voices (51), are characterized by different “tones.” In addition, the configuration of different tones is likely to explain co-occurrences between different clinical conditions and the transdiagnostic nature of SC. From this perspective, we suggest that a useful working hypothesis for future research will include testing whether different types of SC co-occur in comorbid profiles and if specific types of SC can discriminate specific clinical profiles.

### 4.5 Emotional reactions of the self-criticized individual

Models sometimes varied in the descriptions of the emotional reactions experienced by the individual who felt self-criticized. These ranged from feelings of inadequacy to feeling “bad,” with Blatt’s model citing the widest array of emotional responses. Interestingly, in systematic reviews, this central component of SC was greatly overlooked. Indeed, none of the included studies specified the nature of emotional reactions involved in the psychopathological conditions investigated. Consequently, the conceptualization of the role of SC in psychopathological manifestations appeared to suffer from a lack of specification of the emotions involved. Moreover, this lack of clarity makes it hard for researchers to formulate hypotheses regarding the different mechanisms that might link SC to different psychopathological outcomes.

Importantly, these considerations might be linked to the observation that it is not specifically documented which types of emotional reactions are triggered by SC and whether these types are related to specific subdimensions of the process. We suggest that criticism may evolve into different emotional reactions that define the clinical profiles and that the emotional reaction is linked to the individual’s expectations about the environment. Finally, we suggest testing whether different emotional reactions will trigger different action dispositions.

### 4.6 Psychopathological model

At the beginning of this work, we noted that only Blatt’s model was created to explain a single mental disorder (depression), whereas the other models seemed to conceptualize SC as a transdiagnostic factor. However, in the latter, few indications are provided for explaining the moderating variables that might be considered in explanations of why SC leads to a specific clinical condition rather than another. Indeed, to predict developmental trajectories, formulate an accurate prognosis, and well-tailor treatment, it appears necessary to identify which specificities of this transdiagnostic construct account for which specific disorders. Despite several of the retrieved contributions adopting a specific approach (i.e., focusing on only some psychopathological conditions), it is noteworthy that authors did not deepen the discussion of how SC is involved in these specific disorders. These considerations highlight that specific components of SC may not have been deeply analyzed in relation to different psychopathological profiles, whether they are truly transdiagnostic, or whether they are specific to psychiatric disorders. Furthermore, it was not documented if and how SC interacts with other specific psychopathological mechanisms typical of specific psychopathological conditions (e.g., a fear of abandonment).

From the data analyzed and the working hypotheses proposed here, it seems that there are various etiological risk factors that lead to the development of SC processes that can also contribute to defining the emotional tone of criticism and the different emotional reactions that, in turn, are translated into behaviors characterizing different psychopathological manifestations. This stresses the importance of analyzing and explaining this diversity by identifying the different dimensions of SC and classifying them according to the first-discussed components. Beyond the transdiagnostic nature of SC, in clinical practice, one sees how patients criticize and judge their own self negatively according to different criteria or standards, for example: the appearance of non-moral SC through negative evaluations on issues of impotence/inadequacy/incompetence or moral SC through negative evaluations on issues of harmfulness/malice after an immoral use of one’s abilities and the immoral use of one’s own capacities (92–94).

The aforementioned evaluations underlie different emotional reactions. For instance, the fear of deontological guilt (95, 96) of patients with obsessive- compulsive disorder is expressed through a moral critical internal dialogue characterized by anticipated external criticism being a negative self-evaluation of being bad, wrong, and immoral (97). This type of self-talk, characterized by self-blame, is activated when an obsessive thought intrudes, when the individuals consider not implementing compulsion/avoidance, when they judge themselves affected by a mental disorder, or when they fear they committed an unforgivable mistake or caused irreparable harm. Conversely, in some manifestations of depressive symptoms (96), experiences of altruistic guilt are observed (95, 98, 99), associated with sadness elicited by critics regarding the lack of sharing, the lack of compassion, or the belief to have “damaged” the other.

In the cases of social anxiety disorder and eating disorder, criticism is polarized on a non-moral side. It is a self-centered critique regarding themes of incompetence and inadequacy that leads to anxiety, shame, and embarrassment (100, 101). This critique explicates a starting from non-moral reference standards (e.g., intelligence, ability, inclusion, physical prowess, and performative capacity). The same critical connotations can be found in eating disorders.

Finally, per personality disorders, a type of criticism that is often found in patients with borderline personality disorder involves feelings of inadequacy, unworthiness, hate, or disgust toward the self that arise from the reference to a non-moral criterion, regarding inadequacy/incompetence (e.g., lovability, skill, intelligence, aesthetics, performative ability, and inclusion).

Furthermore, the different aspects of SC are likely to be associated with different, or even “pure,” psychological profiles. SC referring to moral criteria may be especially involved in disorders in which guilt plays an important role, such as obsessive–compulsive disorder and depression. On the other hand, to self-criticize one’s own qualities could feel closer to the emotions of shame in, for example, eating disorders or social anxiety. This idea is in line with Miceli and Castelfranchi, (102), who stated the existence of at least two different categories of SC, namely, one pertaining to the moral self and one regarding self-endowment.

Lastly, we suggest that the psychological pain of the individual would be more serious the more structured and the more rigid the person’s positive meta-beliefs regarding criticism are. Believing in the truthfulness, usefulness, and legitimacy of SC would predict a low decentration from problematic mental states and a high negative impact on psychological well-being.

### 4.7 Nature of the variable

We mentioned in *Introduction* that models varied regarding the nature of SC as a psychological variable. This observation was replicated in systematic reviews that differed in conceptualizing SC as a pathological personality trait, a maladaptive and pathological facet, or a vulnerability factor. This evidence suggests the need to clarify the question of whether SC should be conceptualized as a personality trait, a coping strategy, and/or a process. This question should be answered by empirical research through the development of a theoretical model to be tested that contemplates different forms of SC, as previously illustrated.

Furthermore, we argue that it is important to analyze and verify how different forms of SC are related to personality traits or coping mechanisms primarily by studying the correlations from different types of SC and different psychological variables (personality traits, state or trait variables, and mechanisms of coping). Experimental studies testing the effect of state inductions of SC might address this question.

### 4.8 Directions for future research

From the initial evaluation (which made it possible to analyze some key elements and components of SC) of the models, and from the systematic analysis of the authoritative contributions, a weak dialogue emerged between the different theoretical perspectives, which highlighted points of convergence and divergence between authors. The considerations and the theoretical proposals provided identified new questions to be answered by future research framed in integrations of SC that account for the complexity of the construct. This effort could be achieved thanks to the influential conceptualizations by Blatt, Shahar, and Gilbert. Because SC is considered a transdiagnostic construct identifiable in different clinical populations and a negative predictor of psychotherapeutic outcomes, it appears important to reflect on how it can be treated in the different clinical profiles and analyze if specific ingredients, which the literature had not paid attention to, are crucial for discriminating clinical phenotypes and understanding the role of SC in psychopathology.

Therefore, in future studies, it appears fundamental to analyze the different forms of SC and how their contents relate to the various symptoms’ domains. For instance, a few studies have attempted to qualitatively classify the different forms of SC. Some researchers (103), for example, identified three domains of self-criticism (emotional, cognitive, and behavioral), trying to discriminate the concept based on the quality of the criticism. No study, though, attempted to isolate examples of SC to either obtain sub-typologies or test the link between SC and different psychological variables that pertain to psychopathology. No study showed whether different SC can trigger different emotions, thoughts, or behaviors. Expanding this area of investigation calls for studies that deepen the qualitative investigation of SC and how it is modulated in the different clinical phenotypes, questioning if different types of SC account for the interconnection between psychological variables. In particular, it would be important for research to further clarify the connection between SC and specific emotions, particularly guilt and shame. It has been argued that shame elicits self-critical thoughts and the consequent desire to hide or escape from an unpleasant situation, whereas guilt elicits thoughts of inflated responsibility, criticism, and remorse linked to immoral behavior or harm to others (104, 105), even when associated with personal stressful events or psychopathological symptoms (106). An additional and complementarily field of research may also investigate emotional reactions of therapists when encountering patients with high levels of SC (107). Furthermore, it would be crucial to address research regarding the link between SC and psychopathology and extending this investigation to the potential connections with adverse conditions. For instance, a study conducted by Wahyuni et al., (108) in the university population documented the impact of adverse events (i.e., the COVID-19 pandemic) on SC levels, a finding replicated among healthcare professionals exposed to potentially morally injurious events during the pandemic (109). This is an important challenge from an epistemological point of view that might contribute to more effective plans and tailored interventions.

## 5 Conclusion

This meta-review has several limitations. First, we did not examine all the empirical contributions on SC to analyze the theoretical issues related to the construct, focusing only on the contributions that contained the highest levels of evidence available in the scientific research. These contributions are a frequent source of attention and guidance for researchers and clinicians. Second, the preliminary step of identifying the components of the theoretical models may not have been exhaustive, as it was not performed following a validated methodology. Therefore, the conclusions drawn should be considered with caution, although they could be expanded upon by similar studies.

Despite these limitations, this meta-review highlighted that a lack of dialogue between different theoretical perspectives seems to be mirrored by a lack of attention paid to the complexity of the SC construct in key contributions of the field. We suggested some crucial questions to be addressed in order to stimulate knowledge and interest in the multidimensional nature of SC. The results obtained from this study hopefully contribute to the issue of SC in the current scientific landscape.

Shedding light on the weaknesses of the field allowed us to identify new questions and delineate working hypotheses that might stimulate advances in the field by both reducing gaps in the conceptualization and definitions of the construct and examining the specific role of different forms of SC in psychopathology. Thanks to the valuable systematic reviews examined in this work, future researchers aiming to answer the latest questions identified might lead the field to a greater homogeneity in the definition of the construct and shed light on different sub-categories, analyzing how SC declines in the different psychopathological profiles. Recognizing the different aspects or components of SC and, above all, highlighting their special associations with different psychopathologies could have important consequences for psychotherapeutic practice. In particular, it would be important for research to further clarify the connection between SC and specific emotions, particularly guilt and shame. It has been argued that shame elicits self-critical thoughts and the consequent desire to hide or escape from an unpleasant situation, whereas guilt elicits thoughts of inflated responsibility, criticism, and remorse linked to immoral behavior or harm to others (104, 105), even when associated with personal stressful events or psychopathological symptoms (106). An additional and complementarily field of research may also investigate emotional reactions of therapists when encountering patients with high levels of SC (107).

Furthermore, it would be crucial to address research regarding the link between SC and psychopathology and extending this investigation to the potential connections with adverse conditions. For instance, a study conducted by Wahyuni et al., (108) in the university population documented the impact of adverse events (i.e., the COVID-19 pandemic) on SC levels, a finding replicated among healthcare professionals exposed to potentially morally injurious events during the pandemic (109).

## Author contributions

VZ and GR took overall responsibility for the conceptualization and design of the review and revised it critically for important intellectual content. VZ and GR searched for the articles in this review and assessed them for relevance. VZ, GR, and FM were involved in the interpretation of data, in writing and editing the final article, in approving the final version to be published, in agreeing to be accountable for all aspects of the work, and in ensuring that questions related to the accuracy or integrity of any part of the work were appropriately investigated and resolved. All authors contributed to the article and approved the submitted version.
