# Supplementary material for: State of the art of the literature on definitions of self-criticism: a meta-review
Source: Front Psychiatry. 2024 Feb 19;15:1239696. doi: 10.3389/fpsyt.2024.1239696 (PMC10910096; doi:10.3389/fpsyt.2024.1239696)
Supplement: Supplementary file 2 [file DataSheet_2.pdf]

APPENDIX A

Search terms

“SELF-CRITICISM,” “PSYCHOPATHOLOGY,” AND “PSYCHOTHERAPY” - (“SELF CRITI*” OR “SELF-CRITI*”) AND (“PSYCHOPATHOLOGY”) AND (“PSYCHOTHERAPY”) AND (REVIEW" OR "META-ANALYSIS" OR "SYSTEMATIC REVIEW").
